# Structural and Functional Brain Changes beyond Visual System in Patients with Advanced Glaucoma

**DOI:** 10.1371/journal.pone.0105931

**Published:** 2014-08-27

**Authors:** Paolo Frezzotti, Antonio Giorgio, Ilaria Motolese, Alessandro De Leucio, Michele Iester, Eduardo Motolese, Antonio Federico, Nicola De Stefano

**Affiliations:** 1 Department of Medicine, Surgery and Neuroscience, University of Siena, Siena, Italy; 2 Department of Ophthalmology, University of Genoa, Genoa, Italy; Institute Biomedical Research August Pi Sunyer (IDIBAPS) - Hospital Clinic of Barcelona, Spain

## Abstract

In order to test the hypothesis that in primary open angle glaucoma (POAG), an important cause of irreversible blindness, a spreading of neurodegeneration occurs through the brain, we performed multimodal MRI and subsequent whole-brain explorative voxelwise analyses in 13 advanced POAG patients and 12 age-matched normal controls (NC). Altered integrity (decreased fractional anisotropy or increased diffusivities) of white matter (WM) tracts was found not only along the visual pathway of POAG but also in nonvisual WM tracts (superior longitudinal fascicle, anterior thalamic radiation, corticospinal tract, middle cerebellar peduncle). POAG patients also showed brain atrophy in both visual cortex and other distant grey matter (GM) regions (frontoparietal cortex, hippocampi and cerebellar cortex), decreased functional connectivity (FC) in visual, working memory and dorsal attention networks and increased FC in visual and executive networks. In POAG, abnormalities in structure and FC within and outside visual system correlated with visual field parameters in the poorer performing eyes, thus emphasizing their clinical relevance. Altogether, this represents evidence that a vision disorder such as POAG can be considered a widespread neurodegenerative condition.

## Introduction

Glaucoma is one of the leading causes of irreversible blindness worldwide. It is characterized by progressive optic disc cupping, loss of retinal ganglion cells (RGCs) and visual field damage [1]. The most common type of glaucoma in the Caucasian population is the primary open angle glaucoma (POAG), comprising high-tension glaucoma (intraocular pressure [IOP]>21 mmHg) and normal-tension glaucoma (IOP<21 mmHg) [1]. A key role in the pathogenesis of glaucoma is attributed to the RGCs degeneration, which progressively leads to visual field damage evolving, without appropriate therapy, into a bilateral, irreversible blindness [2]. Usually, damage to the axonal projections of the RGCs is reflected by a thinning of the retinal nerve fiber layer of the optic nerve. Recent evidence, however, suggest that abnormalities in glaucoma are not limited to RGCs but extend to the entire visual pathway [3]. In particular, MRI studies, by means of cutting-edge, quantitative techniques such as diffusion tensor imaging (DTI) and voxel-based morphometry (VBM), have shown in POAG the occurrence of damage to the visual system (e.g., reduced microstructural integrity of the optic radiations, decreased volume in visual cortex) [4]–[14]. Furthermore, a recent study using resting functional MRI (fMRI) showed in POAG altered functional connectivity (FC) of the visual network [15]. Histological evidence [16], [17] of neurodegeneration of the visual system in experimental glaucoma supports *in-vivo* MRI data and suggests that MRI-derived findings could reflect the extent of neuroaxonal damage along the entire visual system. However, it is less clear whether brain changes are limited to the visual system and translate into clinically relevant changes. Indeed, if glaucoma is a widespread neurodegenerative disorder, neurodegeneration could occur in the brain similarly to typical neurodegenerative conditions such as Alzheimer disease, amyotrophic lateral sclerosis and Parkinson disease [18]. This supports the notion that visual pathway should not be examined in isolation, but an evaluation of the structural and functional changes across brain would be preferable to properly assess the extent and clinical relevance of neuroaxonal damage in POAG. Against this background, we studied a group of carefully selected patients with an advanced stage of POAG in absence of other ophthalmological and neurological disorders with the aims to ascertain brain changes within and beyond the visual system and to test their clinical relevance. To this end, we exploratively assessed across the whole brain: i) changes in microstructural integrity of white matter (WM) tracts by tract-based spatial statistics (TBSS) of DTI measures, ii) structural changes in grey matter (GM) volume by VBM-style analysis, iii) FC changes of brain networks by resting-fMRI, and iv) how brain changes could be related to visual impairment.

## Materials and Methods

### Study subjects

We enrolled 13 POAG patients (Table 1) and 12 normal controls (NC, 4 males and 8 females, age = 47.3±5.1 years). Patients were recruited among those who were consecutively referring to the Glaucoma Service of the University of Siena and were classified as POAG in presence of typically abnormal optic nerve head, typical glaucomatous visual field loss, open angle at gonioscopy, and no clinically apparent secondary cause for their glaucoma [1]. All patients were on pharmacological treatment for glaucoma. Inclusion criteria for POAG patients were: age between 40 and 70 years, corneal thickness between 520 and 580 µm, glaucomatous damage to the optic nerve and advanced glaucomatous visual field damage according with the Hodapp/Bascom Palmer classification [19]. All patients had visual field defects that were reproducible on multiple repeat testing. Exclusion criteria for POAG patients were: age >70 years, any ocular disorder other than glaucoma, any neurological disorder (documented clinically and instrumentally), use of medications that can affect the visual field, high blood pressure (≥140/90 mmHg), and use of antihypertensive drugs. Subjects in the NC group were recruited among laboratory and hospital workers, had normal neurological and ophthalmological examinations and no history of neurological or ophthalmological disorders. The study received approval from the local Ethics Committee (Azienda Ospedaliera Universitaria Senese). Informed written consent was obtained from all subjects before study entry.

**Table 1:** Clinical-demographic characteristics of our patients with POAG.

*Clinical-demographic characteristics*	*POAG patients*
Age, mean±SD (years)	51.7±6.6
Sex, male/female	10/3
Time from diagnosis (years)	9.4±4.6
Type of poorer performing eye	Right eye (n = 8), left eye (n = 5)
VA of poorer performing eye (decimals)	0.54±0.36
VA of better performing eye (decimals)	0.82±0.32
IOP of poorer performing eye (mmHg)	14.46±4.92
IOP of better performing eye (mmHg)	12.84±2.15
MD of poorer performing eye (dB)	−23.15±7.54
MD of better performing eye (dB)	−9.85±12.31
PSD of poorer performing eye (dB)	9.76±4.09
PSD of better performing eye (dB)	9.2±5.82

See text for abbreviations.

### Visual field measurements

In POAG patients, visual fields were recorded using the Humphrey Field Analyser (Carl Zeiss Meditec, Dublin, CA, USA) running the 30-2 program SITA (Swedish Interactive Threshold Algorithm)-Standard, a standard method for the examination of the 30°central visual field. In this type of measurement, the subject is facing a white illuminated sphere, on which points of light with varying intensities are briefly flashed. Subjects respond when they perceive the flash. The sensitivity at each location in the visual field is determined by changing the intensity of the flash on subsequent presentations. Each eye is measured independently so that one eye is covered while the other is tested. This test provides visual field indices such as: i) Mean Deviation (MD), which refers to the average deviation of sensitivity at each test location from age-adjusted normal population values, providing an indication of the degree of the generalized loss in the visual field; ii) Pattern Standard Deviation (PSD), which is a summary measure of the average deviation of individual visual field sensitivity values from the normal slope after correcting for any overall sensitivity differences.

### MRI acquisition

Brain MRIs were acquired at the NMR Center of the University of Siena using a 1.5 Tesla Philips Gyroscan (Philips Medical Systems, Best, The Netherlands). A sagittal survey image was used to identify the anterior and posterior commissures (AC and PC). Sequences were acquired in the axial plane parallel to the AC-PC line. A dual-echo, turbo spin-echo sequence (repetition time [TR]/echo time [TE]1/TE2 = 2075/30/90 ms, voxel size = 1×1×3 mm) yielded proton density (PD) and T2-W images. DTI data consisted of echo-planar imaging (EPI) (TR = 8500 ms; TE = 100 ms; voxel size = 2.5 mm^3^), with diffusion weighting distributed in 32 directions and b-value = 1000 sec*mm^−2^. The resting-fMRI data were 190 volumes of EPI sequence with TR = 1000 ms, TE = 50 ms, voxel size = 3.75×3.75×6 mm. A high-resolution T1-weighted image (TR = 25 ms, TE = 4.6 ms, voxel size = 1 mm^3^) was acquired for image registration, anatomical mapping and analysis of GM volume.

### MRI and fMRI data analysis

#### Microstructural integrity of the white matter tracts

Voxelwise analysis of DTI data from POAG patients and NC was carried out across the whole brain using TBSS [20] version 1.2, part of the FMRIB Software Library (FSL, www.fmrib.ox.ac.uk/fsl/). First, DTI data were corrected for MRI eddy currents and head motion using affine registration to a reference volume, i.e. the volume without diffusion weighting (b = 0). Second, images of fractional anisotropy (FA), axial diffusivity (AD) and radial diffusivity (RD) were created by fitting a tensor model to the raw DTI data using FDT (FMRIB Diffusion Toolbox) [21] version 3.0, and then brain-extracted using BET (Brain Extraction Tool) [22]. All subjects’ FA data were then aligned into a common standard space (FMRIB58_FA) using the nonlinear registration tool FNIRT (FMRIB Nonlinear Image Registration Tool) [23], [24] which uses a b-spline representation of the registration warp field [25]. Next, the mean FA image was created and thinned to create a mean FA skeleton (thresholded at FA>0.2), which represents the centers of all WM tracts common to the study group, thus avoiding to consider voxels at the edges of the tracts, that may suffer from partial volume effects. Aligned FA data from all study subjects was then projected onto this WM skeleton.

TBSS was also applied to the other DTI-derived data (i.e., AD and RD). In order to achieve this, we used FA images for nonlinear registration, skeletonization and projection stages.

The resulting projected (onto the mean WM skeleton) data of FA, AD and RD images were finally fed into voxelwise group statistics. See Statistics paragraph for details.

#### Grey matter volume

Analysis was performed with FSL-VBM [26] version 1.1, which uses an optimized VBM protocol [27] carried out with FSL tools. First, T1-W images from both study groups (POAG patients and NC) were brain-extracted with BET [22] and GM-segmented [28] before being registered onto the MNI152 standard space using FNIRT [23], [24]. The resulting images were averaged to create a symmetric, study-specific GM template. Second, all native GM images were nonlinearly registered onto this template and “modulated” to correct for local expansion or contraction due to the nonlinear component of the spatial transformation. The modulated GM images were then smoothed with an isotropic Gaussian kernel with a sigma of 3 mm. Because of the use of modulated data, absolute amount (volume) of GM is obtained [27]. See Statistics paragraph for voxelwise analysis of GM volume.

#### Functional connectivity

Analysis was carried out across the whole brain using probabilistic independent component analysis (PICA) [29] as implemented in MELODIC (Multivariate Exploratory Linear Decomposition into Independent Components) version 3.12, also part of FSL.

The following data preprocessing was applied to the input data: motion correction using MCFLIRT [30]; non-brain removal using BET [22]; spatial smoothing using a Gaussian kernel of 8 mm full width at half maximum; normalization of the whole dataset by a single scaling factor (“grand-mean scaling”) in order to ensure dataset comparability at group level; high-pass temporal filtering (Gaussian-weighted least-squares straight line fitting, using a cut-off of 100 s).

In each subject, registration of resting-fMRI data to high-resolution T1-W image and standard space (MNI152) was carried out using FLIRT [30] with BBR (Boundary-Based Registration) cost function and FNIRT (warp resolution = 10 mm) [23], [24]. Pre-processed data were then temporally concatenated across subjects to create a single 4D dataset. Such dataset was variance-normalized and then decomposed into a set of 63 independent components (ICs) using PICA, where the number of dimensions was automatically estimated using the Laplace approximation to the Bayesian evidence of the model order [29]. Next, voxelwise analysis of resting-fMRI data was performed using the “dual-regression” approach [31]. In stage 1, for each subject the group-average set of spatial maps was regressed (as spatial regressors in a multiple regression) into the subject’s 4D space-time dataset. This resulted in a set of subject-specific timecourses, one per group-level spatial map. In stage 2, those timecourses were regressed (as temporal regressors, again in a multiple regression) into the same 4D dataset, resulting in a set of subject-specific spatial maps, one per group-level spatial map.

ICs of interest were selected by visual inspection and by comparison with previously defined resting state networks (RSNs) [32]–[34] and reflect “coactivation” or “synchronization” across the network. The remaining ICs represented physiological noise (cerebrospinal fluid pulsations, head motion), misregistration and scanner artifacts and were thus discarded before further processing.

### Statistics

Differences in age and head movement parameters during resting-fMRI acquisition were tested with Mann-Whitney test. Data were considered significant at p<0.05. SPSS was used to perform such statistical analyses.

As for voxelwise explorative analyses of DTI measures (FA, AD and RD), GM volume and FC, differences between POAG patients and NC and correlation with visual field parameters (MD and PSD) of the poorer performing eye in patients were tested in the general linear model (GLM) framework with, respectively, unpaired t-tests and regression analyses using FSL *randomise*, a nonparametric permutation testing (5000 permutations). The level of significance for all analyses was set at p<0.005, uncorrected, cluster size ≥30 voxels using TFCE (Threshold-Free Cluster Enhancement). Subsequently, in order to further confirm our results we computed within significant clusters mean values across all voxels in each subject and performed comparisons of the different MRI measures between POAG patients and NC with analysis of variance, with Bonferroni correction for multiple comparisons. Moreover, in order to quantify within significant clusters of POAG patients the strength of correlation with MD and PSD of the poorer performing eyes, nonparametric Spearman correlation was used.

To determine whether significant results in resting-fMRI were influenced by brain structural differences across study subjects, GM partial volume was also added to the GLM model as voxel-dependent covariate and voxelwise analyses (both comparisons and correlations) were repeated.

In all analyses, age and sex were used as covariates.

Significant cerebral WM and GM regions were anatomically mapped using standard-space atlases provided by FSL (JHU DTI-based white-matter atlases for WM; Harvard-Oxford cortical structural atlas for GM).

## Results

All patients had bilateral POAG, which was worse in the right eye in eight patients and in the left eye in five patients. In the poorer performing eyes, visual acuity was 0.5±0.3, MD was −23.1±7.5 dB and PSD was 9.7±4.1 dB (Table 1). POAG patients and NC were not different in terms of age (p = 0.15) and, at visual inspection, their brain MRI did not show WM lesions.

### Comparison between POAG patients and NC

#### Microstructural integrity of the white matter tracts

**Figure 1:**
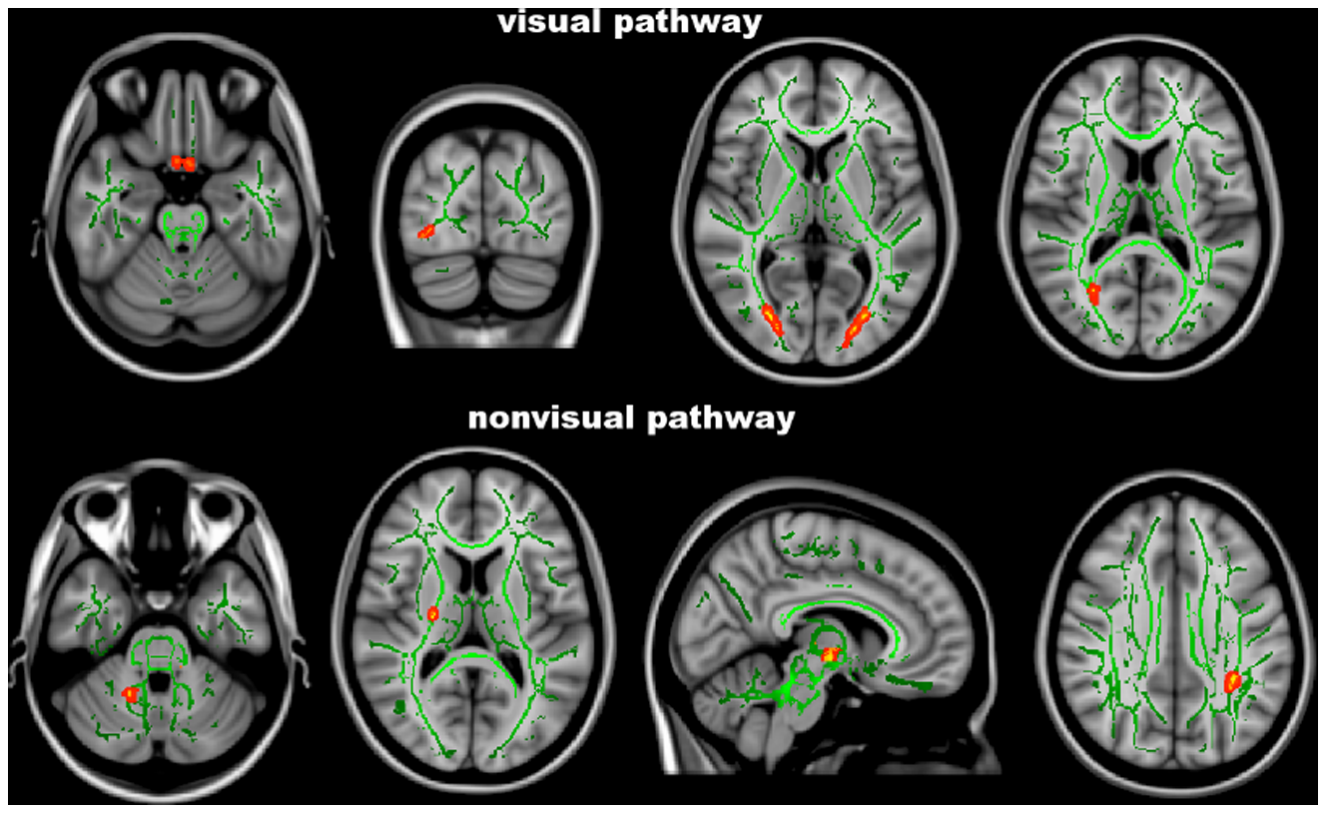
Tract-Based Spatial Statistics analysis. Red-yellow shows clusters where POAG patients have significant DTI abnormalities (lower fractional anisotropy or higher axial/radial diffusivity) in comparison with normal controls. Green is the white matter skeleton. Images (in standard MNI152 space) are shown in radiological convention. See Results and Table 2 for details.

At TBSS analysis across the whole brain (Table 2 and Fig. 1), POAG patients showed lower FA than NC (0.39±0.03 vs. 0.46±0.03, p<0.001) along the visual pathways, namely in the optic tracts/optic chiasm, optic radiations mapping on the right inferior fronto-occipital fascicle (IFOF) and left forceps major (FM), and in the WM adjacent to the left lateral occipital cortex (LOC).

**Table 2:** Regions of the cerebral white matter tracts where patients with advanced POAG showed significant DTI changes with respect to normal controls at whole-brain Tract-Based Spatial Statistics.

*WM regions of the visual pathways* *(local maxima)*	*Side*	*MNI X, Y, Z* *(mm)*	*Cluster size* *(voxel no.)*	*t-value*	*p-value* *
*Lower FA*					
Optic tract	R	5,12, −25	31	4.63	<0.001
Optic tract	L	−9, −1, −14	91	4.36	<0.001
Optic radiation (inferior frontoccipital fascicle)	R	28, −75,4	78	4.93	<0.001
Optic radiation (forceps major)	L	−19, −87,8	228	4.01	0.001
WM of lateral occipital cortex	L	−22, −80,38	52	3.73	<0.001
*Higher AD*					
Optic radiation (inferior longitudinal fascicle)	R	44, −10, −14	468	4.71	<0.001
Optic radiation (inferior frontoccipital fascicle)	L	−34, −24, −5	81	4.82	<0.001
Forceps major adjacent to calcarine cortex	R	26, −63,13	48	3.35	0.001
WM of lateral occipital cortex	R	38, −82, −9	72	4.85	<0.001
*Higher RD*					
Inferior frontoccipital fascicle (adjacent to lingual gyrus)	L	−12, −84, −4	41	4.15	<0.001
***WM regions outside the visual pathways (local maxima)***					
*Higher AD*					
Middle cerebellar peduncle	R	17, −51, −29	46	3.99	0.001
Posterior limb of the internal capsule (corticospinal tract)	R	25, −11,14	164	4.15	<0.001
Anterior thalamic radiation	R	7, −11, −1	35	3.43	<0.001
Superior longitudinal fascicle	L	−31, −47,33	197	4.79	<0.001

*uncorrected.

See text for details and abbreviations.

TBSS analysis of DTI diffusivities confirmed the presence of an altered integrity of the WM tracts in POAG patients with respect to NC (AD: 1.18±0.02 vs. 1.09±0.02×10^−3 ^mm^2^/s, RD: 0.74±0.08 vs 0.63±0.04×10^−3 ^mm^2^/s, p<0.001 for both). Such abnormalities were present, similarly to FA, along the visual pathway: increased AD in the right inferior longitudinal fascicle (ILF), left IFOF, right FM adjacent to calcarine cortex and WM adjacent to the right LOC, and increased RD in the left IFOF, adjacent to the lingual gyrus of the occipital lobe. However, increased AD was found also beyond the visual pathway, namely in the middle cerebellar peduncle, posterior limb of the internal capsule mapping on the corticospinal tract, anterior thalamic radiation on the right, and superior longitudinal fascicle (SLF) on the left.

#### Grey matter volume

**Figure 2:**
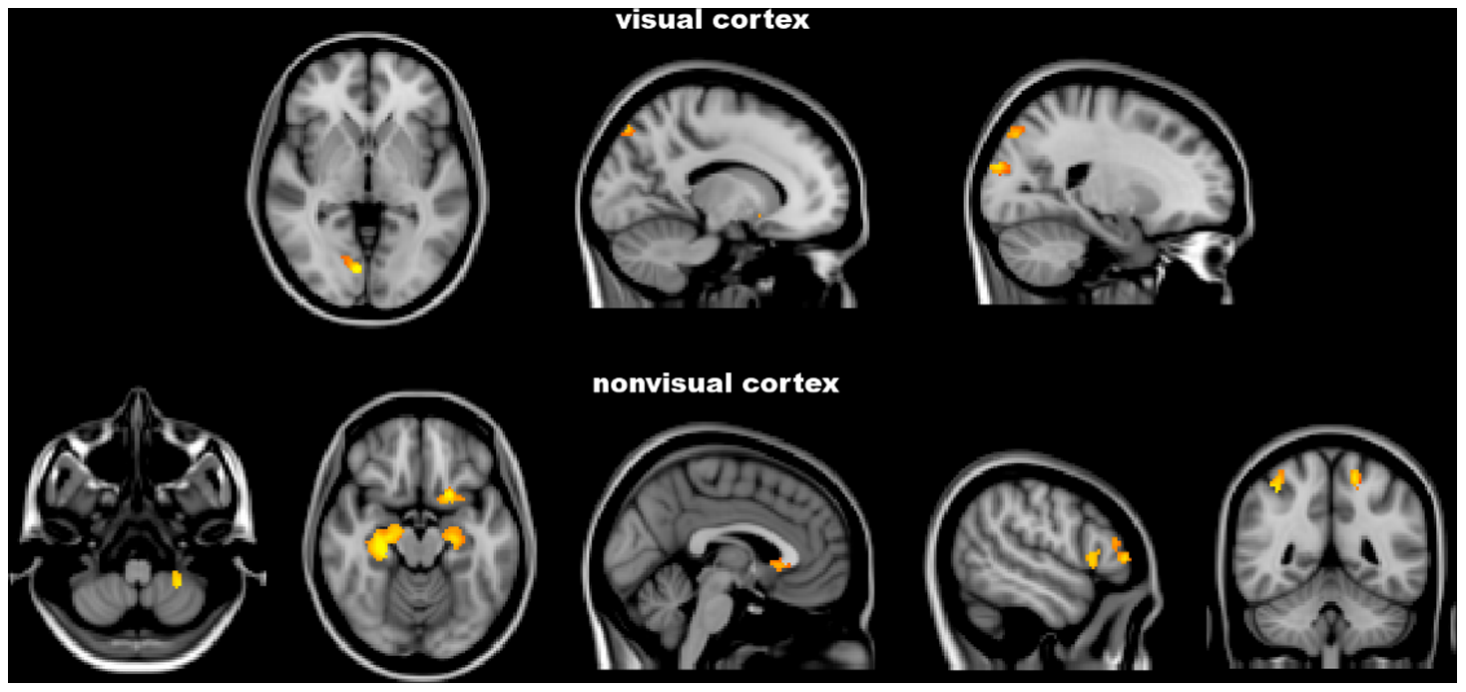
FSL-Voxel Based Morphometry analysis. Red-yellow shows clusters where POAG patients have significantly lower grey matter volume in comparison with normal controls. Images (in standard MNI152 space) are shown in radiological convention. See Results and Table 3 for details.

In several distinct brain regions GM volume, as assessed by a VBM-style analysis (Table 3 and Fig. 2), was lower in POAG patients than in NC (10.43±0.54 cm^3^ vs. 12.28±0.37 cm^3^, p<0.001). This difference mapped on the visual cortex, namely the occipital lobe (lingual gyrus on the right, LOC bilaterally). However, patients also showed GM volume decrease in the cerebellum on the left, temporal lobe (hippocampus bilaterally), frontal lobe (frontoorbital cortex, subcallosal cortex and frontal pole on the left) and parietal lobe (superior parietal lobule on the right, postcentral gyrus on the left).

**Table 3:** Regions of the cerebral grey matter where patients with advanced POAG showed significant decrease in volume (i.e., atrophy) with respect to normal controls at whole-brain FSL-Voxel Based Morphometry.

*GM regions of the visual* *cortex (local maxima)*	*Side*	*MNI X, Y, Z* *(mm)*	*Cluster size* *(voxel no.)*	*t-value*	*p-value* *
Lingual gyrus	R	12, −76, −2	66	5.84	0.001
Lateral occipital cortex	R	24, −80,44	113	4.5	<0.001
	R	22, −86,22	82	4.37	0.001
	L	−18, −80,50	53	4.03	0.001
***GM regions outside the*** ***visual cortex (local maxima)***					
Cerebellum	L	−24, −46, −60	49	4.12	0.002
Hippocampus	R	24, −10, −28	719	4.69	<0.001
	L	−26, −18, −12	451	5.21	<0.001
Frontoorbital cortex	L	−20,16, −18	84	4.57	0.003
Subcallosal cortex	L	−2,24, −2	53	3.30	0.001
Frontal pole	L	−52,42,6	421	3.59	<0.001
Superior parietal lobule	R	28, −56,56	90	3.13	0.001
	R	34, −54,66	88	3.78	0.001
	R	12, −52,76	48	3.22	0.002
Postcentral gyrus	L	−16, −48,56	554	4.13	<0.001

*uncorrected.

See text for details and abbreviations.

#### Functional connectivity

**Figure 3:**
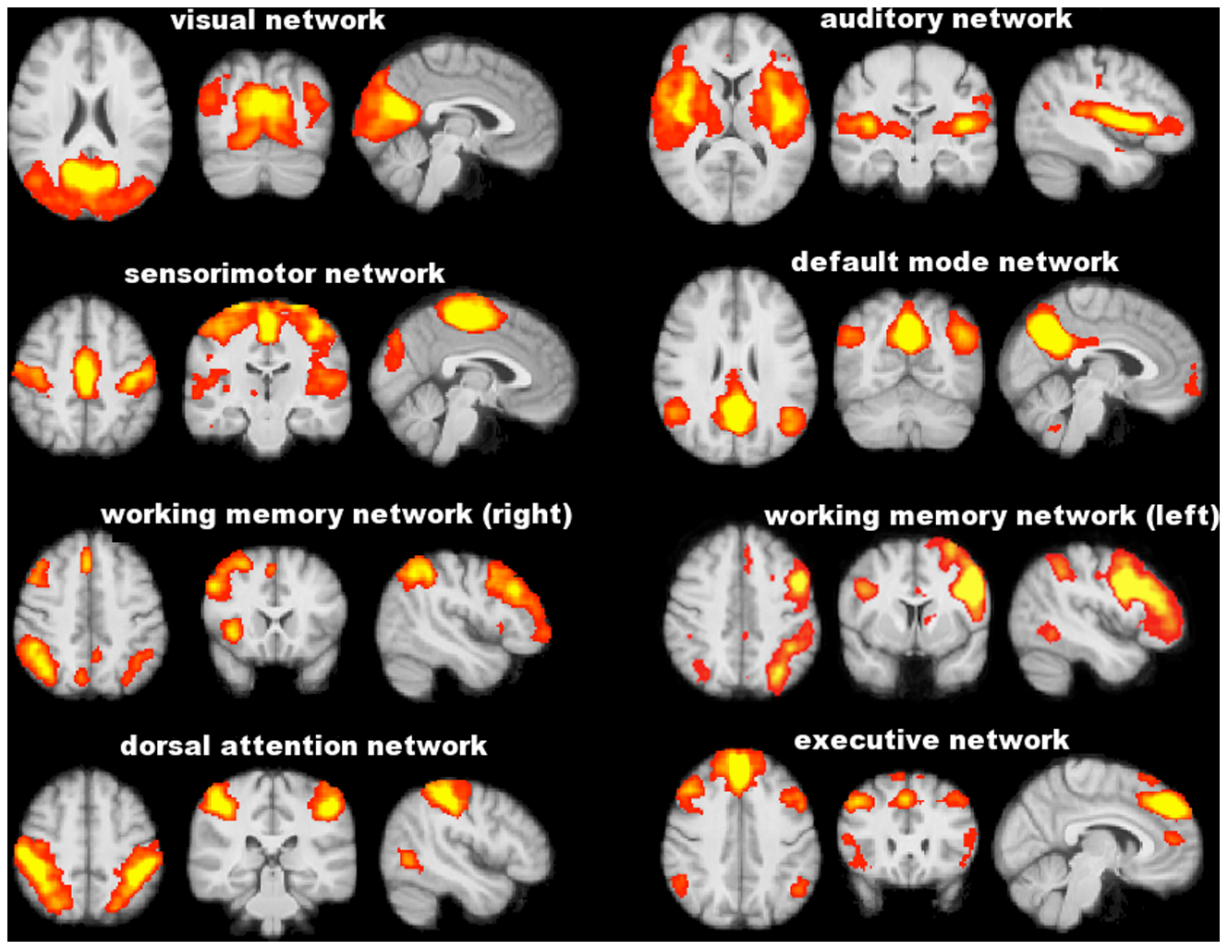
Functionally relevant resting state networks across study subjects (POAG patients and normal controls) identified with independent component analysis. Images (axial, coronal, sagittal) are z-statistics overlaid on the average high-resolution scan transformed into standard (MNI152) space. Red to yellow are z values, ranging from 3 to 10. Images are shown in radiological convention.

**Figure 4:**
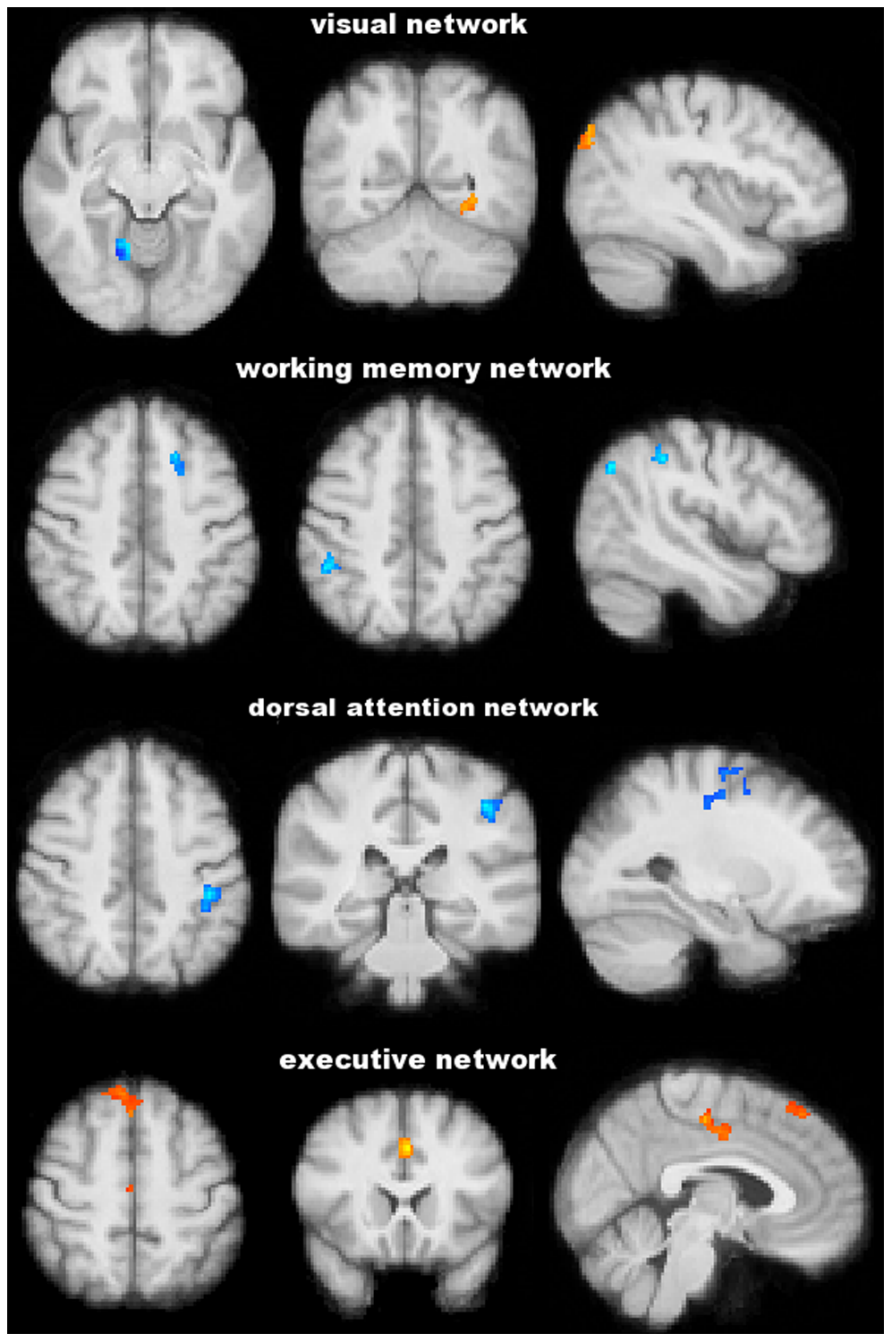
Functional connectivity analysis at the level of resting state networks. Blu-light blu and red-yellow show clusters where POAG patients have, respectively, significantly lower and higher functional connectivity than normal controls. Images (in standard MNI152 space) are shown in radiological convention. See Results and Table 4 for details.

No difference was found between POAG patients and NC in the head movement parameters during resting-fMRI acquisition (relative displacement: 0.08±0.05 mm vs. 0.06±0.02 mm, p = 0.90; absolute displacement: 0.26±0.06 mm vs. 0.27±0.13 mm, p = 0.57). PICA across the whole brain of the study population defined eight functionally relevant RSNs (Fig. 3), including visual network, auditory network, sensorimotor network, default mode network, working memory network (right and left), dorsal attention network and executive network. Five of these RSNs showed significant differences in terms of FC between POAG patients and NC (Table 4 and Fig. 4). In particular, POAG patients had lower FC in an extrastriate region of the visual network (lingual gyrus on the right) (0.72±0.85 vs. 1.70±0.68, p = 0.016), in the working memory network (superior frontal gyrus on the left [0.25±0.44 vs. 1.26±0.72, p<0.001]; supramarginal gyrus and LOC on the right [1.10±0.54 vs. 2.44±0.36, p<0.001]) and in the dorsal attention network (LOC bilaterally, pre- and postcentral gyrus on the left) (0.71±0.33 vs. 1.68±0.31, p<0.001). Conversely, POAG patients had higher FC than NC in the visual network (LOC bilaterally and temporo-occipital fusiform cortex on the left) (1.60±0.70 vs. 0.94±0.44, p = 0.007) and in the medial part of the executive network (superior frontal gyrus, parancigulate gyrus and anterior cingulate) (0.87±0.31 vs. 0.13±0.28, p<0.001). Because of the differences in GM volume between POAG patients and NC (see the above paragraph), we repeated the FC comparison analysis also adding GM volume as a voxelwise covariate to the GLM design. After such correction, FC differences in the above RSNs were all retained.

**Table 4:** Grey matter regions of the resting state networks where patients with advanced POAG showed significant changes in functional connectivity with respect to normal controls at probabilistic Independent Component Analysis across the whole brain.

*GM regions of the visual network* *(local maxima)*	*Side*	*MNI X, Y, Z* *(mm)*	*Cluster size* *(voxel no.)*	*t-value*	*p-value* *
*Lower FC*					
Lingual gyrus	R	14, −58, −8	46	4.13	<0.001
*Higher FC*					
Lateral occipital cortex	R	36, −86,20	66	4.16	<0.001
	L	−38, −82,32	60	3.04	<0.001
Temporo-occipital fusiform cortex	L	−24, −56, −12	46	3.84	<0.001
***GM regions outside the visual network (local maxima)***					
*Lower FC*					
Working memory network					
Superior frontal gyrus	L	−18,22,46	94	6.11	<0.001
Supramarginal gyrus	R	48, −42,44	76	4.75	<0.001
Lateral occipital cortex	R	48, −68,38	72	4.85	<0.001
Dorsal attention network					
Lateral occipital cortex	R	28, −76,28	335	4.19	<0.001
	L	−42, −80, −10	309	4.16	<0.001
	L	−30, −84,2	81	3.3	<0.001
	R	44, −66, −10	42	2.91	<0.001
Precentral gyrus	L	−34, −12,60	205	3.73	<0.001
Postcentral gyrus	L	−40, −36,46	154	5.45	<0.001
*Higher FC*					
Executive network					
Superior frontal gyrus	R	4,38,50	109	3.85	<0.001
Paracingulate gyrus	M	0,20,34	56	6.24	<0.001
Anterior cingulate	R	4, −18,48	146	4.36	<0.001

*uncorrected.

See text for details and abbreviations.

### Relationship between brain changes and visual field parameters in the poorer performing eyes

**Figure 5:**
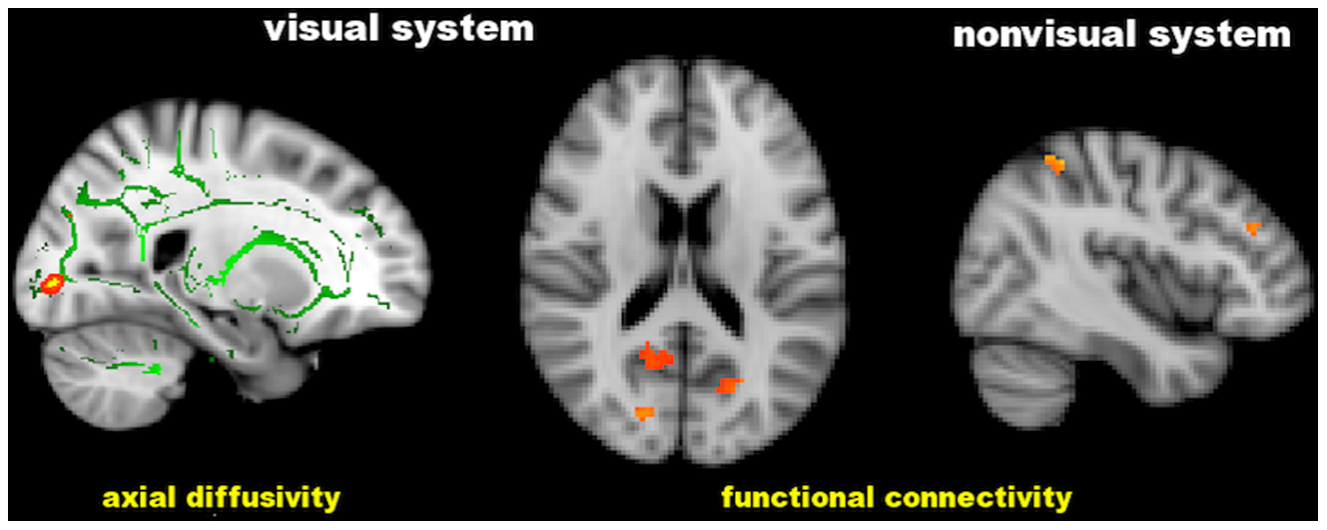
Significant correlation of the visual field Mean Deviation with cerebral axial diffusivity and functional connectivity in distinct brain regions of patients with POAG. Green is the white matter skeleton. Images (in standard MNI152 space) are shown in radiological convention. See Results and Table 5 for details.

Lower MD values correlated with changes in the visual system (Table 5 and Fig. 5): higher AD in the WM of the right occipital pole (r = −0.67, p = 0.01) and lower FC in the visual network (precuneous bilaterally, calcarine cortex on the right and cuneal cortex on the left) (r = 0.48, p = 0.045). Lower MD values also showed correlation with changes outside the visual system (Table 5 and Fig. 5), namely with lower FC in the working memory network (inferior frontal gyrus on the left, middle frontal gyrus and superior parietal lobule on the right) (r = 0.50, p = 0.045).

**Table 5:** Brain regions where our patients with advanced POAG showed significant correlations with visual field Mean Deviation values in the poorer performing eyes.

*Regions of the visual system* *(local maxima)*	*Side*	*MNI X, Y, Z* *(mm)*	*Cluster size* *(voxel no.)*	*t-value*	*p-value* *
*Higher AD in visual pathway*					
WM of occipital pole	R	23, −90, −1	37	3.83	0.001
*Lower FC in visual network*					
Precuneous	L	−8, −56,10	37	2.78	<0.001
	R	16, −52,14	114	2.01	<0.001
Calcarine	R	8, −76,24	53	2.0	<0.001
Cuneal cortex	L	−8, −76,26	67	2.0	<0.001
***Regions outside the visual system (local maxima)***					
*Lower FC in nonvisual networks*					
Working memory network					
Middle frontal gyrus	R	46,36,28	30	2.1	0.001
Superior parietal lobule	R	46, −46,56	49	2.47	<0.001

*uncorrected.

See text for details and abbreviations.

**Figure 6:**
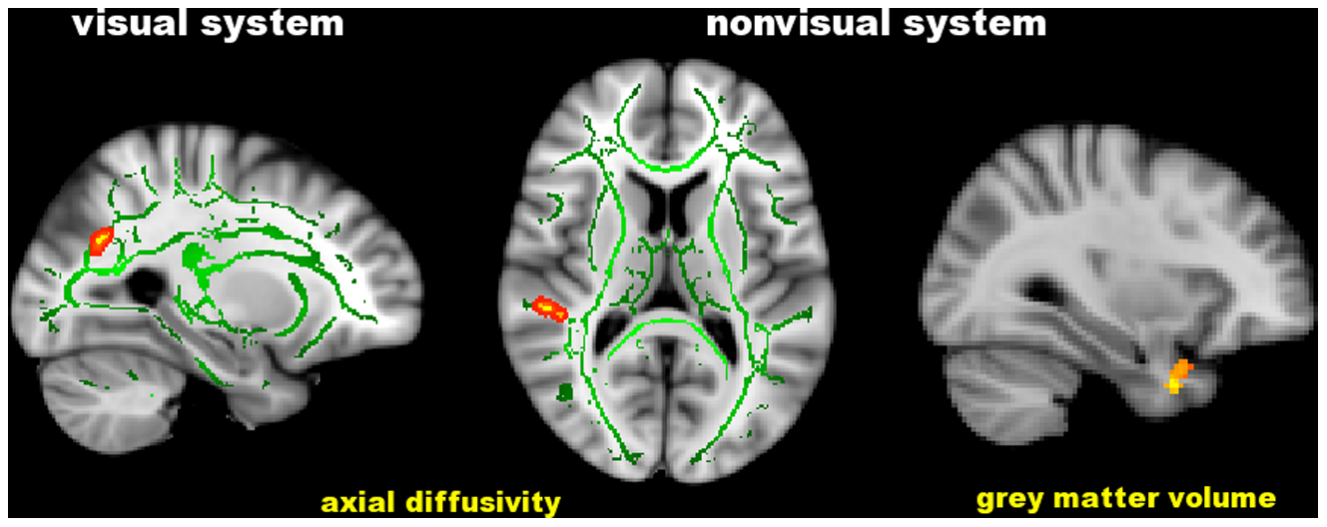
Significant correlation of the visual field Pattern Standard Deviation with cerebral axial diffusivity and grey matter volume in in distinct brain regions of patients with POAG. Green is the white matter skeleton. Images (in standard MNI152 space) are shown in radiological convention. See Results and Table 6 for details.

As regards the PSD (Table 6 and Fig. 6), higher values correlated with higher AD in the WM of LOC (r = 0.52, p = 0.04), which is part of the visual system and with structural changes in nonvisual regions: increased AD in the WM of the planum temporale on the right (r = 0.52, p = 0.04) and decreased GM density in the temporal pole on the right (r = −0.55, p = 0.03). In all the FC correlation analyses, significant clusters were retained after adding GM volume as a voxelwise covariate.

**Table 6:** Brain regions where our patients with advanced POAG showed significant correlations with visual field Pattern Standard Deviation values in the poorer performing eyes.

*Regions of the visual system* *(local maxima)*	*Side*	*MNI X, Y, Z* *(mm)*	*Cluster size* *(voxel no.)*	*t-value*	*p-value* *
*Higher AD in visual pathway*					
WM of lateral occipital cortex	L	−28, −66,23	59	4.3	<0.001
***Regions outside the visual system (local maxima)***					
*Higher AD in nonvisual pathway*					
WM of planum temporale	R	51, −31,10	32	4.02	<0.001
*Lower density in nonvisual GM*					
Temporal pole	R	28,14, −48	84	4.8	0.001

*uncorrected.

See text for details and abbreviations.

## Discussion

Evidence of neurodegeneration along the visual pathway in human and experimental glaucoma has been reported in several *in-vivo* and *ex-vivo* studies [35]. While this is presumed to be a type of secondary anterograde trans-synaptic degeneration primed by the death of RGCs, a primary neurodegenerative process has not been excluded. Moreover, whether this process remains confined to the visual system or tends to spread trans-synaptically to other brain systems is currently unknown. This latter issue can be addressed with the use of novel MRI-based quantitative techniques enabling the study of structural and functional connectivity across brain. Thus, in the present exploratory study, we used them to ascertain in a small group of carefully selected patients with advanced POAG and without other ophthalmological and neurological disorders whether brain changes could be found not only within, but also beyond the visual system.

### Structural brain changes

Microstructural integrity of the WM tracts and volume of the GM turned out to be altered in our POAG patients in most of the visual system. In the WM, this comprised IFOF and ILF: the former reaches the superior frontal cortex rostrally, dorsal parietal and occipital cortex caudally, all regions involved in visuospatial function; the latter connects the occipital and the anterior temporal cortices, playing a role in visual memory. GM atrophy was found here in the most anterior and medial parts of the visual cortex (anterior lingual gyrus), as previously described in POAG [4] and also in the most posterior and lateral regions (LOC). Moreover, a recent study found reduced cortical thickness in primary and secondary visual cortex in a heterogeneous group of POAG patients [36]. Interestingly, some of the altered WM tracts connected regions of the visual cortex showing GM atrophy, supporting the presence of a trans-synaptic degenerative process as the responsible for the intimate pathological mechanism of POAG. Other WM tracts and cortical GM regions that are not part of the typical visual pathway were altered in our POAG patients. Indeed, abnormalities were found in WM tracts somehow related with visual processing such as the SLF, which provides the prefrontal cortex with information regarding perception of visual space and supplies the parietal cortex with information on focusing spatial attention and regulating selection and retrieval of spatial information. However, also WM tracts that are unrelated with the visual system (i.e., anterior thalamic radiation, corticospinal tract and middle cerebellar peduncle) proved to be altered in our study. In addition, POAG patients showed GM atrophy in regions involved in cognitive processing such as the hippocampus (memory), frontoorbital cortex (decision-making) and superior parietal lobule (spatial orientation). Interestingly, many of these structures are crucially involved in the tau pathology of Alzheimer disease, trans-synaptically spreading from the limbic structures to other regions of the brain [37]. The significant topographic relationship between visual field indices and many of these brain regions also emphasizes the clinical relevance of these findings.

### Functional brain changes

In addition to brain structural abnormalities, we also found in our POAG patients FC changes in different RSNs. In patients with severe impairment of visual field, decreased FC in visual and working memory networks could be interpreted as due to maladaptation, thus contributing to clinical deficits. Indeed, the correlation of lower FC in both these networks with worst visual field lends support to this hypothesis. Some of the regions from the two networks where we found decreased FC (lingual gyrus, pre- and postcentral gyri) were, alongside primary visual cortex, suggested to be associated in normal people with consolidation of visual memory [38]. Decreased FC in the working memory may reflect reduced integration of visual information and may explain the impairment of object identification in POAG patients. The working memory network actually corresponds to the dorsal visual stream, which is part of the neural processing of vision (“how pathway”) engaged in processing spatial location of objects (spatial awareness) in order to program behavior. Functionally, the dorsal visual stream contains a detailed map of the visual field and it is able to detect and analyze movements in the nearby space. On the other hand, we also found in our POAG patients increased FC in the visual and executive networks. The increased FC we found in the visual association (secondary) cortex (LOC and temporo-occipital fusiform cortex) may be due to a loss of functional inhibition caused by damage to the visual input traveling along the visual pathway and towards the primary visual cortex. The increased FC within the executive network mapped on the medial frontal cortex, which has a role in monitoring actions and performance outcomes in goal-directed behavior. It is possible that FC increase in patients with reduced visual acuity, such as POAG, is due to compensatory mechanisms. Indeed, the occurrence of compensatory cortical plasticity after elevated IOP, demonstrated in a primate model of glaucoma, is in line with this interpretation [39].

### Glaucoma as neurodegenerative condition

The findings of widespread brain abnormalities observed in the present study delineate POAG as a more complex disorder than classically thought, capable of involving, at least in advanced stages, unanticipated brain structures and functions. In support of this theory, anatomical structural network analysis of human brain [40] demonstrates the presence of connections among distinct regions of the visual and nonvisual cortex that showed atrophy in our POAG patients. In particular, the right lingual gyrus is connected with the LOC bilaterally and with the ipsilateral parahippocampal cortex, superior parietal cortex and left frontal pole. Moreover, the LOCs in both hemispheres are connected between them and with lingual gyrus but also with the right superior parietal cortex, left postcentral gyrus and frontal pole. As such, glaucoma could be interpreted as the expression of a complex neurodegenerative process at cerebral level [41] and its diffuse involvement of different distinct structures and functions could be expression of the spread of neurodegeneration, similarly to what is found in typical neurodegenerative conditions such as Alzheimer disease, amyotrophic lateral sclerosis and Parkinson disease [18]. In line with this hypothesis, there is evidence indicating a close link between glaucoma and neurodegenerative conditions on the basis of the similarities in the loss of selective neuron populations, in the trans-synaptic disease spreading from injured neurons to connected and distant neurons, and in the common mechanisms of apoptosis, including oxidative injury, glutamate excitotoxicity and abnormal protein accumulation [41]. Indeed, experimental work suggests the presence in glaucoma of a neuronal alteration of the constitutive autophagy process, a molecular mechanism also present in Alzheimer disease and that could cause accumulation of misfolded proteins (e.g., β-amyloid and tau in Alzheimer disease) as well as templating and spreading of proteins across brain, ultimately leading to neural cell death [18]. Finally, in addition to this experimental evidence, the prevalence of glaucoma was found to be much higher in different cohorts of patients with Alzheimer disease than in NC and this was independent from the IOP values [42]–[44].

It must be stressed here that, in POAG, mechanisms other than trans-synaptic degeneration can potentially cause diffuse brain abnormalities. It is well known that most of the visual pathways are located within watershed areas of increased vulnerability to ischemic damage. Indeed, patients with glaucoma may have a silent cerebral small vessel disease, characterized by the presence of WM lesions and the absence of focal neurological symptoms [45]. However, because of our careful selection of patients with advanced POAG and without clinical and instrumental (including WM lesions) evidence of other ophthalmological and neurological conditions, a vascular insult is less likely to have caused such widespread brain abnormalities. In addition, atrophy of the visual-related GM can be caused by a chronic reduction of visual input. However, the presence of a widespread (i.e., well beyond the visual system) and not localized GM atrophy and the absence of regions with compensatory increased GM volume, as locally found in a previous VBM study on advanced POAG [46], make the process of cortical structural plasticity less likely as explanation of our findings. As such, our results and those reported above corroborate the hypothesis that glaucoma can be considered a progressive neurodegenerative condition potentially spreading throughout the brain.

### Study strengths and limitations

Different MRI technologies were applied here to study both structure and FC across brain. Indeed, we applied a robust and sensitive analysis approach, which allows exploring abnormalities across the entire brain rather than limiting the analysis on predefined regions, as previously done in POAG. Such an approach thus ensured an independent assessment of brain structures and networks unrelated to the visual system.

Possible limitations lie in the uncorrected voxelwise thresholding, in the cross-sectional design and in the small sample size. Whole-brain voxelwise analyses were performed with an uncorrected statistical threshold of p<0.005, as the results did not survive more conservative corrections for multiple comparisons. Such thresholding may provide an “explorative” overview of brain damage and its clinical relevance in POAG patients. In order to alleviate false positive rate, we required significant clusters to have a relatively stringent extent threshold of at least 30 neighboring voxels, similarly to the approach used for explorative analyses by several other studies [47]–[52]. Moreover, following a previous approach [53], a full Bonferroni correction was used in the comparison analyses performed on the regions-of-interest of the significant clusters derived from the uncorrected voxelwise statistics. It is important to stress here that voxelwise statistical inference was performed in this study using a nonparametric permutation testing, which relies on minimal assumptions and can be applied when the assumptions of a parametric approach are untenable, as it happens in the case of small study groups.

As for the small sample size, we preferred to consecutively recruit a selected group of patients with advanced POAG and without other ophthalmological and neurological disorders, in order to allow an appropriate assessment of the neurodegeneration spreading. Importantly, this selected group of POAG patients was compared with a group of age-matched NC, but, given the relatively advanced age of both groups, FC data were corrected for GM volume, thus ensuring that the reported changes are exclusively due to FC abnormalities.

### Conclusions

By using a multimodal MRI approach, this exploratory study demonstrates the presence in patients with advanced POAG of structural and functional changes that go well beyond the visual system, suggesting that POAG can be considered a vision disorder falling within the group of neurodegenerative disorders [41] and, as such, spreading throughout the brain [18]. Larger, longitudinal studies covering the whole range of clinical deficits in POAG are warranted in order to confirm and extend this observation, which may have relevant implications for understanding the complex clinical scenario in glaucoma and may encourage new therapeutic strategies in this progressively disabling disorder.
